# Supplementary material for: The Zinc Finger Protein Zfp2 Regulates Cell–Cell Fusion and Virulence in Cryptococcus neoformans
Source: J Fungi (Basel). 2025 Dec 7;11(12):868. doi: 10.3390/jof11120868 (PMC12734157; doi:10.3390/jof11120868)
Supplement: Supplementary file 1 [file jof-11-00868-s001.zip › jof-3980817-supplementary/supplementary material/Figure S1 and S2.pdf]

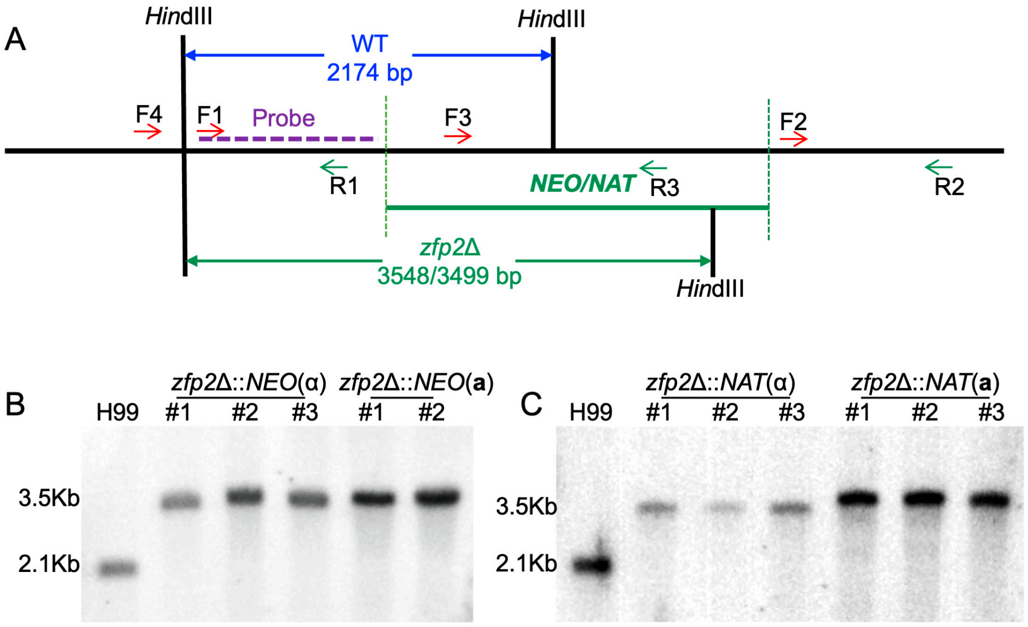

Figure S1. Verification of *zfp2*Δ mutants by Southern blot. (**A**) Genomic DNA was digested with restriction enzymes for Southern blot analysis. The PCR products from TL837/TL838 (F1/R1) serve as templates for probe synthesis. The wild-type strain H99 shows a 3.5-Kb band, while the *zfp2*Δ mutants display a 2.1-Kb band. (**B**) Southern blot analysis of *ZFP2*-disrupted transformants. All genomic DNA samples were digested with *Hin*dIII, separated by gel electrophoresis, and hybridized with a probe targeting the upstream flanking sequence of *ZFP2*, as shown in (A). Consistently, a 2.1-Kb band was detected in *zfp2*Δ mutants, whereas a 3.5-Kb band appeared in the wild-type strain H99.

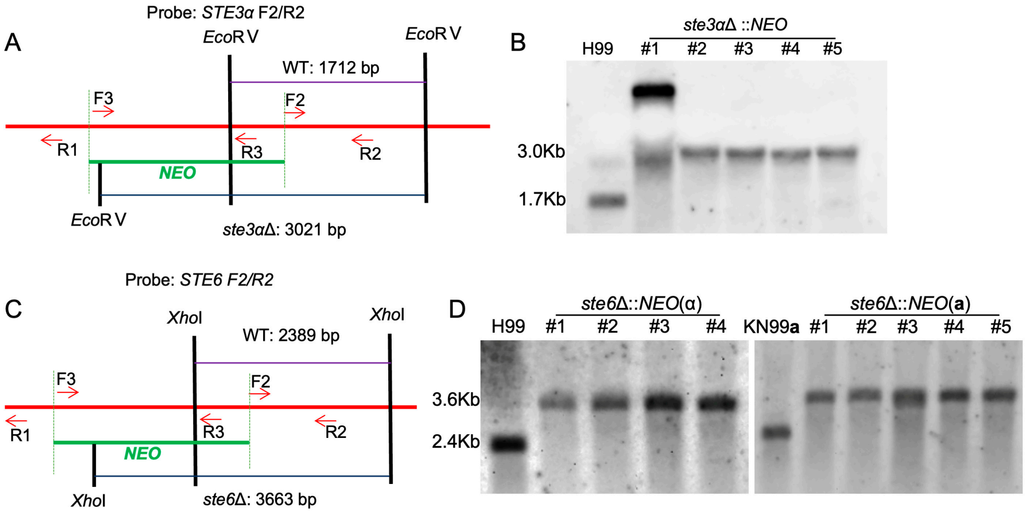

Figure S2. Verification of the ste3*α*Δ and ste6Δ mutants using Southern blot. (**A**) Restriction enzymes digest genomic DNA for Southern blot analysis. The TL1515/TL1516(F2/R2) PCR products serve as templates for probe synthesis. The wild-type strain H99 produces a 1.7-Kb band, while the ste3*α*Δ mutants produce a 3.0-Kb band. (**B**) Southern blot analysis of *STE3α*-disrupted transformants. All genomic DNAs were digested with *Eco*RV, separated by electrophoresis, and hybridized with a probe located in the downstream flanking sequence of *STE3α*, as shown in (A). As expected, a 3.0-Kb band was detected in *mfα1*Δ mutants, in contrast with a 1.7-Kb band in the wild-type strain H99. (**C**) Restriction enzymes cut genomic DNA for Southern blot analysis. The TL1508/TL1509(F2/R2) PCR products are used as templates for probe synthesis. The wild-type strains H99 and KN99**a** show a 2.4-Kb band, while the ste6Δ mutants show a 3.6-Kb band. (**D**) Southern blot analysis of *STE6*-disrupted transformants. All genomic DNAs were digested with *Xho*I, separated by electrophoresis, and hybridized with a probe located in the downstream flanking sequence of *STE6*, as shown in (C). As expected, a 3.6-Kb band was detected in ste6Δ mutants, compared to a 2.4-Kb band in the wild-type strain H99 or KN99**a**.
