# Supplementary material for: The Zinc Finger Protein Zfp2 Regulates Cell–Cell Fusion and Virulence in Cryptococcus neoformans
Source: J Fungi (Basel). 2025 Dec 7;11(12):868. doi: 10.3390/jof11120868 (PMC12734157; doi:10.3390/jof11120868)
Supplement: Supplementary file 1 [file jof-11-00868-s001.zip › jof-3980817-supplementary/supplementary material/Table S1 Strains and plasmids used in this study.pdf]

Table S1. Strains and plasmids used in this study

Strains/plasmids	Genotype/properties	Source/reference
E. coli		
DH5α	cloning strain	
C. neoformans		
H99	*MAT*α	Perfect et al., 1993
KN99**a**	*MAT***a**	Nielsen et al., 2003
YSB119	*MAT*α *NAT*	Bahn et al., 2004
YSB121	*MAT***a** *NEO*	Bahn et al., 2004
TBL237	*MAT*α *zfp2*Δ*::NEO*	In this study
TBL298	*MAT***a** *zfp2*Δ::*NEO*	In this study
TBL383	*MAT*α *zfp2*Δ::*NAT*	In this study
TBL384	*MAT***a** *zfp2*Δ::*NAT*	In this study
TBL305	*MAT*α *zfp2*Δ::*NEO ZFP2*::*NAT*	In this study
TBL359	*MAT***a** *zfp2*Δ::*NEO ZFP2*::*NAT*	In this study
TBL344	*MAT*α *zfp2*Δ::*NEO* P_{H3}-*GFP*-Zfp2::*NAT*	In this study
TBL345	*MAT***a** *zfp2*Δ::*NEO* P_{H3}-*GFP*-Zfp2::*NAT*	In this study
TBL386	*MAT*α *zfp2*Δ::*NEO* P_{ACTIN}-Zfp2-HA::*NAT*	In this study
TBL365	*MAT***a** *zfp2*Δ::*NEO* P_{ACTIN}-Zfp2-HA::*NAT*	In this study
TBL454	*MAT*α *CNAG_06808*(*STE3α*)*::NEO*	In this study
TBL462	*MAT*α *CNAG_03600*(*STE6*)*::NEO*	In this study
TBL463	*MATa CNAG_03600*(*STE6*)*::NEO*	In this study
Plasmids		
pCN19	Amp^r Plasmid harboring *GFP* under histone H3 promoter	Price et al., 2008
pTBL1	Amp^r Plasmid harboring *NAT* marker	Fan et al., 2019
pTBL183	Amp^r Vector for P_{ZFP2}-*ZFP2-NAT* for *ZFP2* complementation	In this study
pTBL211	Amp^r Vector for P_{Actin}-*ZFP2-HA-NAT* for *ZFP2* overexpression	In this study
pTBL195	Amp^r Vector for P_{H3}-*GFP-ZFP2-NAT* for Zfp2 localization	In this study
