# Supplementary material for: The Zinc Finger Protein Zfp2 Regulates Cell–Cell Fusion and Virulence in Cryptococcus neoformans
Source: J Fungi (Basel). 2025 Dec 7;11(12):868. doi: 10.3390/jof11120868 (PMC12734157; doi:10.3390/jof11120868)
Supplement: Supplementary file 1 [file jof-11-00868-s001.zip › jof-3980817-supplementary/supplementary material/Table S2 PCR primers used in this study.pdf]

Table S2. PCR primers used in this study

Primers	Description	Sequence (5'-3')
TL17	M13F	GTAAAACGACGGCCAG
TL18	M13R	CAGGAAACAGCTATGAC
TL19	*NEO* split F	GGGCGCCCGGTTCTTTTTGTCA
TL20	*NEO* split R	TTGGTGGTCGAATGGGCAGGTAGC
TL59	*NEO* R4	TGTGGATGCTGGCGGAGGATA
TL217	*GAPDH* qRT-PCR F	TGAGAAGGACCCTGCCAACA
TL218	*GAPDH* qRT-PCR R	ACTCCGGCTTGTAGGCATCAA
TL837	*ZFP2* KO F1	CCTGCGGCCGTCTTATTTGGTTTA
TL838	*ZFP2* KO R1	CTGGCCGTCGTTTTACAGGTGGTGGTCGACGCTCTCC
TL839	*ZFP2* KO F2	GTCATAGCTGTTTCCTGGACCGGCGCCAGCTCGTATCGT
TL840	*ZFP2* KO R2	TTCGGCCGCCTGTCTCATCAATCT
TL841	*ZFP2* KO F3	CTTTGCCATTGCTCCCCATTCGTA
TL842	*ZFP2* KO R3	GCGGTTCGTGCCTATCGGTCCTG
TL843	*ZFP2* KO F4	CTTTTTGGCTGCGGTCTTGTGCTA
TL1098	*ZFP2* Comp F1	CGGTGGCGGCCGCTCTAGAACTAGTGGATCTTCGGCCGCCTGT CTCATCAATCT
TL1099	*ZFP2* Comp R1	CGCCCAACATGTCTGGATCCATGTGGTAAGCCTTATCTTTGAA A
TL1100	*ZFP2* HA (Actin) F1	CGCCCAACATGTCTGGATCCATGTGGTAAGCCTTATCTTTGAA A
TL1101	*ZFP2* HA (Actin) R1	ACGTCGTATGGGTAGGATCCTCTTCTCTCCTTGGCGTTCAGCTG
TL1124	GFP-Zfp2 F1	GACGAGCTGTAcGGATCCATGTGGTAAGCCTTATCTTTGAAA
TL1125	GFP-Zfp2 R1	CTGGCGGCCGTTACTAGTTTATCTTCTCTCCTTGGCGTTCAG
TL1279	*ZFP2* Q-PCR F1	GGAGATGGCTTTCCTGTGTAA
TL1280	*ZFP2* Q-PCR R1	GACTCCTCTCTGGTTTCTCTTTG
TL1494	*MFα1* Q-PCR F1	CTTCACTGCCATCTTCACCA
TL1495	*MFα1* Q-PCR R1	ACACAAAGGGTCATGCCA
TL1496	*STE3*α Q-PCR F1	CCGTGCAATTCGGGCTAAA
TL1497	*STE3*α Q-PCR R1	CCGAGACGGCAACCATTAT
TL1500	*STE6* Q-PCR F1	GCGGACAAGCTCAAAGAT
TL1501	*STE6* Q-PCR R1	CCTCCTGTACTCTACCTTCTC
TL1513	*STE3*α KO F1	TCGGGGGAGTAAGAAGAGACAG
TL1514	*STE3*α KO R1	CTGGCCGTCGTTTTACGAGGGAAAAGAAAACAAAAAGACATT
TL1515	*STE3*α KO F2	GTCATAGCTGTTTCCTGCGCTACGTAATCAAACTCCAATCAG
TL1516	*STE3*α KO R2	GCCCTACCCCGCTCTACCA
TL1517	*STE3*α KO F3	TTTTTCGTTGATGGCATTGTCTGGT
TL1518	*STE3*α KO R3	TCTCGGCGGAAAGGTAAAA
TL1519	*STE3*α KO F4	TTCAACACCCTTAAGGCAGCATAC
TL1506	*STE6* KO F1	GGCGTCCGTACCGTCACCAGT
TL1507	*STE6* KO R1	CTGGCCGTCGTTTTACGGCGGCTCCTTCCATAATCA
TL1508	*STE6* KO F2	GTCATAGCTGTTTCCTGGAAGACGGGAGAATGGGAGTAAAT
TL1509	*STE6* KO R2	AGAAGAGGAAGGGCAAAAGTAAGG
TL1510	*STE6* KO F3	CGCGCCACTGCTGCAATCTCTAC
TL1511	*STE6* KO R3	ATCGCCAATCGCTGCCTCTGTCCT
TL1512	*STE6* KO F4	GCAAGGTCCAAGCCAACGATACG
